# Supplementary material for: Molecular characterization of CHAD domains as inorganic polyphosphate-binding modules
Source: Life Sci Alliance. 2019 May 27;2(3):e201900385. doi: 10.26508/lsa.201900385 (PMC6537752; doi:10.26508/lsa.201900385)
Supplement: Supplementary file 1 [file LSA-2019-00385_TableS1.pdf]

Table S1 - Crystallographic data collection and refinement.

	RcCHAD	CtCHAD – apo (sulfate)	CtCHAD – polyP
**PDB-ID**	**6QV5**	**6QV7**	**6QVA**
**Data collection**			
Space group	$P\,6_1\,2\,2$	$I\,2\,2\,2$	$I\,2\,2\,2$
Cell dimensions			
a, *b*, *c* (Å)	116.4, 116.4, 119.3	69.5, 95.7, 102.8	69.4, 97.0, 106.4
α, β, γ (°)	90, 90, 120	90, 90, 90	90, 90, 90
Resolution (Å)	41.65 – 2.30 (2.44 – 2.30)	36.80 – 1.72 (1.82 – 1.72)	19.70 – 2.05
$R_{meas}^{\#}$	0.172 (3.68)	0.074 (2.21)	0.155 (2.35)
CC(1/2)$^{\#}$	0.99 (0.50)	1.0 (0.50)	1.0 (0.43)
$I/\sigma\,I^{\#}$	15.5 (1.0)	16.0 (1.0)	14.1 (1.1)
Completeness (%)$^{\#}$	99.9 (99.6)	100 (99.8)	99.6 (99.6)
Redundancy$^{\#}$	21.2 (21.5)	7.0 (7.1)	13.3 (13.2)
Wilson B-factor$^{\#}$	62.5	39.3	46.1
**Refinement**			
Resolution (Å)	41.65 – 2.30	36.8 – 1.72	19.70 – 2.05
No. reflections	21,737	70,305	21,397
$R_{work/}\,R_{free}^{\$}$	0.232 / 0.242	0.189 / 0.222	0.208 / 0.255
No. atoms			
protein	2,237	2,584	2,518
ligand	5	88	71
solvent	27	179	68
Res. B-factors$^{\$}$			
protein	84.2	42.6	49.7
ligand	74.8	71.6	98.2
solvent	56.0	51.4	49.5
R.m.s deviations$^{\$}$			
bond lengths (Å)	0.011	0.01	0.01
bond angles (°)	1.49	1.07	1.73
Ramachandran plot$^{\$}$:			
most favored regions (%)	98.25	99.36	98.70
outliers (%)	0	0	0
MolProbity score$^{\$}$	1.25	1.24	1.01

Highest resolution shell is shown in parenthesis.

[*]as defined in XDS (Kabsch, 1993)

[+]as defined in autoBUSTER (Global Phasing Limited) (RcCHAD), phenix.refine (Adams *et al*, 2010) (CtCHAD apo) or Refmac5 (Murshudov *et al*, 1997) (CtCHAD - polyP)

[$]as defined in Molprobity (Davis *et al*, 2007)
